# What Do We Know about Spondylodiscitis in Children? A Retrospective Study

**DOI:** 10.3390/children9081103

**Published:** 2022-07-22

**Authors:** Ayla Yagdiran, Charlotte Meyer-Schwickerath, Raphael Wolpers, Christina Otto-Lambertz, Katrin Mehler, Andre Oberthür, Nikolaus Kernich, Peer Eysel, Norma Jung, Kourosh Zarghooni

**Affiliations:** 1 Department of Orthopedic and Trauma Surgery, Faculty of Medicine and University Hospital of Cologne, University of Cologne, 50937 Cologne, Germany; raphael.wolpers@outlook.com (R.W.); christina.otto-lambertz@uk-koeln.de (C.O.-L.); nikolaus.kernich@uk-koeln.de (N.K.); peer.eysel@uk-koeln.de (P.E.); kourosh.zarghooni@uk-koeln.de (K.Z.); 2 Department I of Internal Medicine, Faculty of Medicine and University Hospital of Cologne, Center for Integrated Oncology, University of Cologne, 50937 Cologne, Germany; charlotte.meyer-schwickerath@uk-koeln.de (C.M.-S.); norma.jung@uk-koeln.de (N.J.); 3 Department of Pediatrics, Faculty of Medicine and University Hospital of Cologne, University of Cologne, 50937 Cologne, Germany; katrin.mehler@uk-koeln.de (K.M.); andre.oberthuer@uk-koeln.de (A.O.)

**Keywords:** pediatric spondylodiscitis, clinical course, diagnosis, treatment, predicting factors

## Abstract

Pediatric spondylodiscitis (PSD) is a rare disease with a major impact on mobility and functional status. Data concerning demographic and microbiological characteristics, clinical course, treatment, and outcome are scarce. Therefore, the aim of this study was to present clinical experiences of a third-level hospital (2009–2019) in PSD and compare these with adult spondylodiscitis (ASD). Of a total of 10 PSD patients, most of the infants presented with unspecific pain such as hip pain or a limping, misleading an adequate diagnosis of spine origin. Eight patients could be treated conservatively whereas surgery was performed in two cases with one case of tuberculous PSD (tPSD). The causative agent was detected in three of the patients. The diagnosis of PSD is often difficult since clinical symptoms are unspecific and causative pathogens often remain undetected. Nevertheless, empirical anti-infective therapy also seems to be effective. Based on recent studies, clinicians should be encouraged to keep the duration of anti-infective therapy in children short. Since comorbidities are not presented in PSD it is unclear which children suffer from PSD; thus, studies are necessary to identify predisposing factors for PSD. In our study, PSD differs from ASD in diagnostic and especially in therapeutic aspects. Therefore, specific guidelines for PSD would be desirable.

## 1. Introduction

Pediatric spondylodiscitis (PSD) is a rare disease involving spondylodiscitis and vertebral osteomyelitis, with or without an associated soft-tissue abscess, mainly in psoas muscle [1]. In the past, spondylodiscitis (SD) and vertebral osteomyelitis were considered as two separate diseases depending on the bony involvement. Nowadays, the two entities are considered to be different stages of the same pathological process assigned to the generic term PSD [2]. Due to the limited data information on PSD incidence is estimated to be in the range of 0.3 per 100.000 [3]. Nevertheless, the prevalence of PSD has increased over the past few years [4]. Methicillin-sensitive Staphylococcus aureus (*MSSA*) is the most common pathogen causing PSD [3,4,5,6]. Similar to SD in adults, an early diagnosis and treatment are crucial for treatment success in PSD [4,7]. Due to its rarity and non-specific presentation, which is mainly attributed to the inability of younger children to verbalize symptoms and/or locations the diagnosis and treatment of PSD are often delayed [8]. Further complicating factors are the low sensitivity of blood cultures and CT-guided biopsy showing negative results in 92% and 52%, respectively [5]. PSD can require prolonged hospitalization and antibiotic use leading to lost school days. However, unnecessary diagnostic tests and considerable healthcare expenditure should be avoided. In most cases, a PSD heals non-surgically with anti-infective therapy and without long-term consequences [9]. Surgical treatment is required in more extensive infections causing vertebral instability and/or neurological impairment [1]. In fact, there is a lack of consensus on diagnosis and treatment approaches in PSD. Unlike in adult SD (ASD) [10], no guidelines supported by clinical evidence are available on the management of PSD, including the diagnostic workflow and type, route and duration of antibiotic therapy [1,2,6].

The aim of this study was to analyze data on PSD presentation, diagnostics, and treatment in a pediatric third-level center over a period of 10 years. Secondly, to compare principles of PSD with ASD. We hypothesized that the guidelines of ASD cannot be readily applied to PSD.

## 2. Materials and Methods

### 2.1. Patient Selection

The hospital database was searched for patients aged between 0 and 18 years with diagnosis of discitis and SD between January 2009 and December 2019. Based on the international classification of diseases (ICD) 10th revision the code M46. was used. All charts of the children identified by the search were reviewed by a pediatric infectious disease’s specialist and an orthopedist with pediatric expertise. All imaging (Magnetic Resonance imaging = MRI, Computer Tomography = CT, X-ray, and Sonography) had been conducted and/or assessed by a pediatric radiologist. After an extensive chart review and an interdisciplinary reevaluation of the diagnosis four children were identified not to fulfill the criteria for a PSD.

### 2.2. Definition

Inclusion criteria were based on the definition of PSD by Fernandez et al. Children were considered if they presented with clinical findings compatible with PSD with abnormal radiographic images [11]. MRI is the mainstay of diagnosis showing involvement of the disc. Consequently, PSD was diagnosed by a multidisciplinary team (pediatric infectious disease’s specialist, pediatric orthopedic and a radiologist with expertise in children) in children with low-grade clinical infection and disc inflammation in MRI.

### 2.3. Data Collection

In this retrospective study we report detailed information on the demographic and microbiological characteristics, clinical course, and treatment of all cases of PSD in a third- level center. The following demographic and clinical parameters were collected: epidemiological data (age and sex), medical history (comorbidities, immunosuppression, and history of trauma), and clinical manifestations (pain and location, fever (>38.5 °C), limping, refusal to walk, neurological impairment, time between clinical onset and establishing the diagnosis), complications (paraspinal abscess, psoas abscess, vertebral body destruction) and PSD site.

The following diagnostic parameters were recorded: laboratory values at admission (erythrocyte sedimentation rate = ESR, normal value: <20 mm/h; white blood cell = WBC count; C-reactive protein = CRP, normal value: <5 mg/L; and hemoglobin = Hb, normal value: >10 g/dL), imaging (sonography, X-ray, CT, MRI), microbiological findings by culture or broad range polymerase chain reaction (PCR) assay (bacteremia, blood culture, CT-guided biopsy, intra-operative taken biopsy and tests for tuberculosis). In addition, these treatment informations were listed: type of treatment (non-surgical or surgical), length of anti-infective therapy (intravenous (iv), oral (os), and total), and duration from admission until initiation of therapy.

Part of the data are used in another publication comparing antibiotic treatment for pediatric bone and joint infection before and after the implantation of a pediatric infectious disease consultation service.

### 2.4. Ethics

The study was approved by the ethics committee of our institution (file number: 20-1120) and complies with the principles of the Declaration of Helsinki (1996).

## 3. Results

In total, 14 children with suspected PSD were retrieved from the hospital database and the diagnosis of PSD was confirmed in 10 children. Every patient received infectious disease consultation to diagnose SD and exclude other infections not related to this episode.

Epidemiological data and clinical manifestations at admission are shown in Table 1.

The majority of the patients were boys (*n* = 7) and the median age was 5 varying between 8 months and 17 years. In total, 70% of the children were under 4 years.

None of the children had predisposing factors such as immunodeficiency or comorbidities. Three children had a history of trauma at presentation.

All children presented with pain; however, different locations of pain were mentioned: five patients suffered from back pain, four from hip pain, and one from abdominal pain. Further, seven children refused to walk, six showed limping on admission, one child had a history of fever, and none had neurological impairment.

Median time between clinical onset and establishment of the diagnosis was 9.2 weeks and varied between 1 and 52 weeks.

The lumbar spine was affected in most of the cases (*n* = 9/10).

Complications, as a whole, were found in 8/10 patients (also combined): in particular, the destruction of the vertebral body was found in 7 cases, the abscess of the psoas muscle in 4, and the paraspinal abscess in 3.

Laboratory data, microbiological findings, and treatment of PSD are presented in Table 2.

The results of laboratory data at admission showed elevated ESR levels in 5 cases and slightly elevated CRP levels in 6 out of 10 cases. All children had normal WBC counts and one child had decreased Hb.

**Figure 1:**
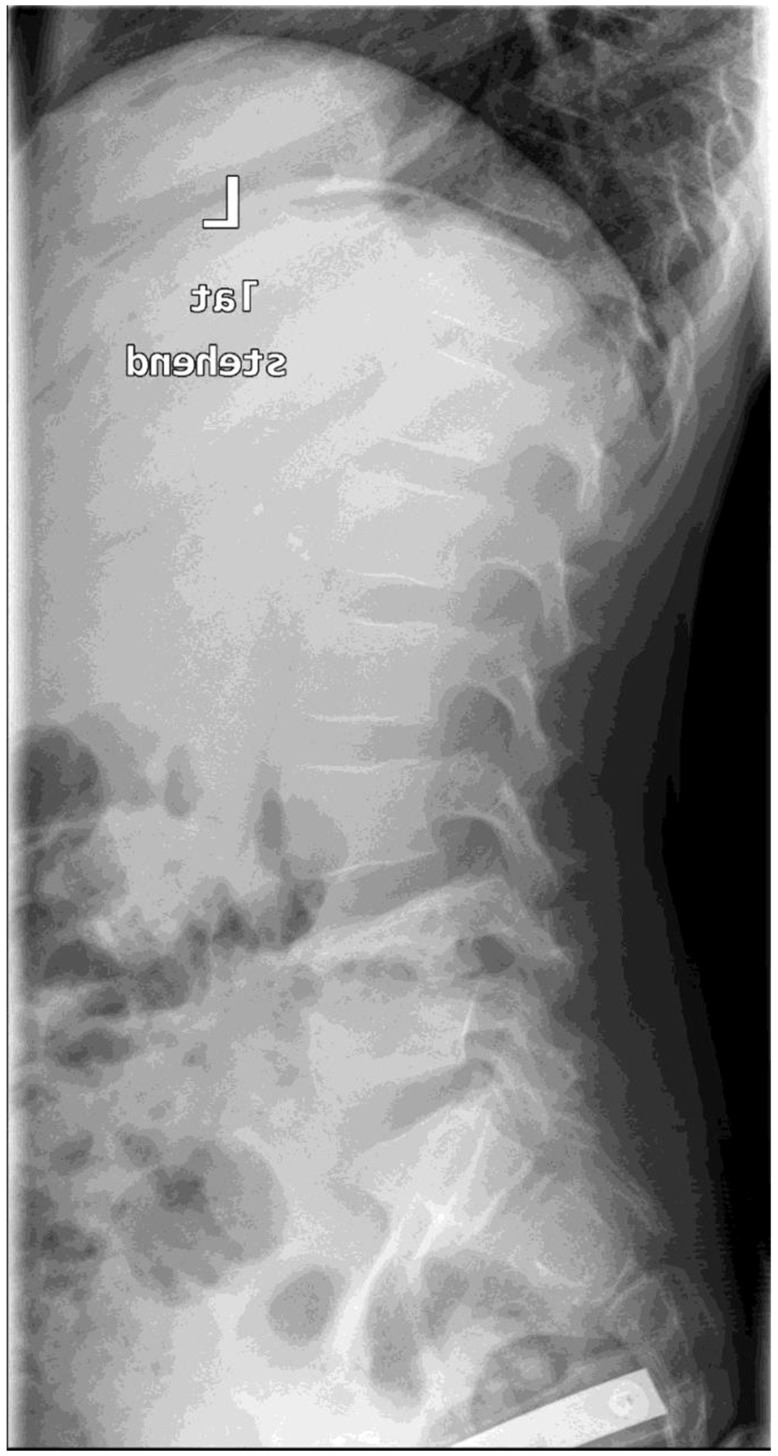
X-ray (sagittal view) of the lumbar spine (Pat. No. 10).

**Figure 2:**
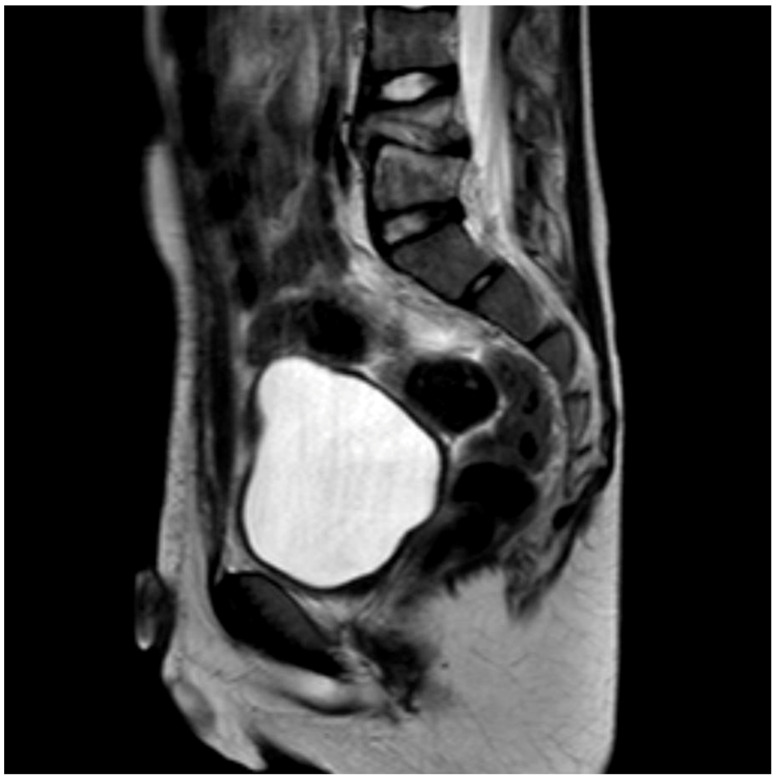
MRI scan (sagittal view) of the lumbar spine (Pat. No. 10).

Different methods of imaging were initiated before detecting the final diagnosis of PSD: five children received a sonography of the hips and four patients an X-ray image (Figure 1) while in two cases a CT was performed. The final diagnosis was made by MR-imaging in all cases (Figure 2).

Blood cultures were taken during the diagnostic workup in the majority of the cases (*n* = 8/10). Although none of the children received anti-infective therapy at the time of taking the blood cultures, no case revealed a positive result. In six cases, tuberculosis tests were performed. The final pathogen was detected only in three cases.

Only two children were surgically treated, one of those being infected by Mycobacterium tuberculosis and the other one infected by *MSSA* and being the oldest (17 years) in the cohort. Both were treated conservatively at the beginning but received surgical treatment in the course due to treatment failure.

Intravenous antibiotics were administered in all cases except the tuberculosis case and duration in most cases varied between 2 and 4 weeks, followed by an oral regimen which also varied between 2 and 4 weeks. One child with *MSSA* SD received only intravenous antibiotics for 6 weeks.

Most of the children (*n* = 7) were treated with antibiotics for 6 weeks, only one child received a 4-week treatment. The patient with tuberculosis was treated for a distinct longer period (21 months). One patient was lost to follow up due to a transfer to another hospital.

The time from admission until initiation of therapy varied between 1 and 16 days.

## 4. Discussion

We present a comprehensive retrospective analysis of PSD patients who needed hospitalization in a tertiary care hospital focusing on clinical characteristics during the last 10 years. Our main findings were as follows: (i) Children aged under 4 years are at risk of developing a PSD. (ii) While half of the patients suffer from back pain (50%), a high proportion (40%) complain about hip pain rather than back pain causing additional examinations. (iii) Although vertebral destruction is often present, most of the PSD patients are treated successfully non-surgically.

### 4.1. Epidemiological Data and Clinical Manifestations 

Baseline clinical characteristics in our cohort study were in line with other studies [2,5,6]. PSD predominantly affected boys (7/10) in our cohort. Moreover, PSD was mostly diagnosed in lumbar spine (9/10). The majority of the cases were located in L5/S1 (4/10). The most commonly reported symptoms were refusal to walk (70%), limping (60%), and back pain (50%). No comorbidities were detected. Except for limping/refusal to walk and the lack of comorbidities, these findings are also in accordance with ASD [10].

A recent multicenter study by Dayer et al. showed a biphasic rather than a triphasic distribution of PSD with a higher incidence of the infantile group with children aged between 6 months and 4 years (79%) and a smaller peak (20%) in the juvenile group aged between 5 and 18 years [5]. Our study is in agreement with this observation as we were also able show a biphasic PSD distribution with mostly infantile patients (70%) and a smaller proportion of juvenile patients (30%). This age-dependent distribution of symptoms and etiology of PSD may support the former distinction between SD occurring mainly in children aged < 5 years and vertebral osteomyelitis which is more common in older children from a clinical point of view [5].

Several studies pointed out that due to the rarity and non-specific presentation with pain of PSD, whose nature and location is inadequately described by infants, the diagnosis of PSD is often missed, with consequent delay of treatment and development of sequelae [1,2,3,4,6]. The median time to reach the diagnosis in our cohort was 9.2 weeks, ranging from 1 to 16 weeks, out of the child with tuberculous PSD (tPSD) who required almost 1 year. This time is in agreement with that reported by Kang et al. [3]. In ASD it can take 12 weeks to get a diagnosis, too [10]. Due to its specificity of presentation, diagnosis and treatment, tPSD deserves attention among PSD although Germany is not an endemic region. In our study, tPSD occurred at the age of 8 years, which is not typical for tPSD [1].

Generally, in PSD pain and movement limitation prevailed over other signs of inflammation (e.g., fever) at onset. Only one patient had fever at admission

### 4.2. Medical History

Strikingly, no child of our series had any comorbidity or showed immunodeficiency at presentation. This finding is contrary to the adult population, where number and severeness of present comorbidities is not only a risk but also a predictive factor of poor prognosis [12,13].

### 4.3. Complications

Complications in PSD and ASD are divided into two types of origin: infectious and neurological. The overall rate of complications in our PSD cohort was 50%, which is comparable to the cohort of Cavalieri et al. [6]. Interestingly, all complications had an infectious origin and no neurological etiology. On the contrary, neurological impairment occurs in every fifth patient in ASD [14].

### 4.4. Laboratory Results

The results of laboratory test related to a primary pyogenic spinal infection (WBC, CRP, and ESR) provide limited information, since they are normal or only slightly elevated [5,6]. Accordingly, our study presents children with modest clinical and biological inflammatory responses to infection. All children had normal WBC counts. CRP level was slightly elevated in 6 out of 10 cases and, therefore, seems to be a less sensitive marker of inflammation in PSD compared to data in ASD [10]. Strikingly, five children showed elevated ESR levels. In a retrospective cohort study, Cavalieri et al. presented increased ESR level as the most frequent inflammatory PSD marker at diagnosis which is paralleled by our cohort [6].

### 4.5. Imaging

For musculoskeletal pain, the gold standard is plain radiography in two planes as the first imaging method [15]. Thus, the adequate diagnostic tool (MRI of the spine) was not considered immediately. This may be attributed to the pain which was often (50%) located outside of the spine region. Furthermore, a MRI is elaborate to perform as the children often require sedation or anesthesia. Typical MRI alterations are a reduced disc height, disc hypointensity on T1-weighted images, and disc hyperintensity on T2-weighted images. The involved disc shows fluidlike signal intensity in T1- and T2-weighted MRI images.

Moreover, limping and refusal to walk regularly prompted sonography of the adjacent joint which is typical for children, since the pain often radiates to the immediate regions and coxitis fugax is the most common differential diagnosis in toddlers [16].

A CT scan was only performed in cases when an MRI was not able to distinguish infection and tumor.

### 4.6. Microbiological Testing

The results of blood and tissue cultures are usually the only means of anti-infective treatment. In ASD, the proof of a causal pathogen is to be aimed for in any case for the goal of an effective and efficient anti-infective therapy. Thus, pathogen detection is successful in the majority of the cases (67–100%) [10] in ASD. Blood cultures are positive in 58% in ASD [10]. In contrast, blood cultures in PSD are usually sterile and only positive in 8% [5]. In our study, none of the blood cultures taken at presentation were positive, even though none of the patients received anti-infective therapy before sampling. In the past, the standard in our clinic was trying to detect the pathogens in PSD to adapt a specific anti-infective therapy. Therefore, four children underwent a CT-guided needle biopsy recording one positive case (Patient No.2). This finding is agreement with Afshari et al. who also identified a low rate of positive cultures (20%) [17]. This is comparable to ASD, where CT-guided needle biopsy is able to detect the causing pathogen in 19–30% [10]. Nowadays, we only carry out invasive investigations on children who fail to improve with anti-infective treatment or when the presence of atypical organisms is suspected. Taking into account all diagnostic measures for pathogen identification (blood cultures, CT-guided needle biopsies, and samples during surgery), we were able to identify the causative pathogen in 3 out of 10 cases). A precise statement on the pathogens of PSD is not possible, as most cultures were negative. In the culture positive cases, Staphylococcus aureus was the prevalent etiological agent (2/10), which is in line with the literature, both in PSD and ASD [2,5,10].

### 4.7. Treatment

As the microbiological diagnostic yield is low in PSD, empirical anti-infective treatment has to be administered mostly. Since data related to the duration of anti-infective treatment of PSD are rare, the current pediatric practice is derived from the management of ASD and relies upon the experience of the treating clinicians. Thus, in our study empirical anti-infective therapy was administered based on our local standard regimen for adults with spondylodiscitis. As MRSA rates are below 10% in Germany [18], we prefer the combination of cefotaxime and flucloxacillin. This is not consistent with the Bone and Joint Infection Guidelines (ESPID Guidelines) [19]. Since empirical anti-infective therapy differs widely in Europe the European Society for Pediatric Infectious Diseases (ESPID) published guidelines in 2017 to offer a consensus-based practice recommendation: in low-risk settings most suggest monotherapy with an antistaphylococcal Penicillin, Clindamycin, or a 1st or 2nd generation cephalosporin.

In our cohort, anti-infective therapy was given routinely for 2 weeks intravenously, followed by a highly bioavailable oral anti-infective therapy. Generally, the total duration of the anti-infective treatment was 6 weeks and was shortened or extended depending on the individual interdisciplinary decision. Due to lacking data, no defined criteria are established for oral switch and treatment termination. In addition, most authors agree upon long periods of anti-infective therapy in PSD [2,6]. Accordingly, in our study antibiotic regimen, oral step down and length of treatment vary. Although Roversi et al. were able to show that a prolonged anti-infective therapy in PSD does not affect the outcome, neither in a positive nor in a negative way [1].

In 2016, Mc Mullen et al. published a systematic review on antibiotic duration for numerous bacterial infections in children including guidelines for pediatric bone and joint infections [20]. Additionally, the ESPID guideline [19] and more recently country-specific consensus guidelines on the treatment of osteomyelitis in children were published [21,22]. These studies are helpful in the attempt to shorten the duration of anti-infective therapy in children, although data on PSD are lacking.

Due to the severe spinal deformity causing chronic pain, defined as pain lasting longer than 3 months, two of the PSD patients (one caused by *MSSA* one by mycobacterium tuberculosis) were operated on. In a recent systematic review on 340 patients, the rate of surgery was 6% [2]. Interestingly, both PSD patients who received surgery had a history of trauma. Ferri et al. reported a rate of 6% of trauma in history in PSD but unfortunately without any further data concerning the treatment associated with this specific occasion [2].

Our study had a hypothesis generating character. Thus, the limited number of patients may not capture the full scope of possible clinical experience in PSD. A second potential limitation is the single-center design of the study. As clinical standards might differ between clinics or regions, results and conclusions might also vary in disparate clinical contexts. Nevertheless, our study gives important clinical data specific to PSD differing from ASD. Therefore, guidelines established for adults cannot be easily applied to children.

## 5. Conclusions

The diagnosis of PSD is often difficult since clinical symptoms are unspecific and causative pathogens often remain undetected. Nevertheless, empirical anti-infective therapy also seems to be effective. Based on recent studies, clinicians should be encouraged to keep the duration of anti-infective therapy in children short. Since comorbidities are not presented in PSD it is unclear which children suffer from PSD; thus, studies are necessary to identify predisposing factors for PSD.

In our study, PSD differs from ASD in diagnostic and especially in therapeutic aspects. Therefore, specific guidelines for PSD would be desirable.

## Figures and Tables

**Table 1:** Epidemiological data and clinical manifestations at admission.

Patient	1	2	3	4	5	6	7	8	9	10
Age in years (months)	1 (22)	16	3	2	1 (15)	0 (8)	0 (10)	2	17	8
Sex	m	m	f	m	m	f	m	m	f	m
**Medical history:**										
Comorbidities	no	no	no	no	no	no	no	no	no	no
Immunosuppression	no	no	no	no	no	no	no	no	no	no
Trauma	no	no	no	no	no	no	no	yes	yes	yes
**Clinical manifestations:**										
Pain	yes	yes	yes	yes	yes	yes	yes	yes	yes	yes
Location of pain	hip	spine	hip	spine	legs	legs + hip	abdominal	hip + spine	spine	spine
Fever (>38.5 °C)	no	no	no	no	no	no	no	yes	no	no
Limping	yes	no	yes	yes	yes	yes	no	yes	no	no
Refusal to walk	yes	yes	yes	yes	yes	yes	no	yes	no	no
Neurological symptoms	no	no	no	no	no	no	no	no	no	no
Time elapsed between clinical onset and establishing the diagnosis (weeks)	1.5	3	2.5	8	3	3	3	1	1	52
PSD site	L5/S1	L2/3	L3/4	L5/S1	L5/S1	L2/3	L2/3	L5/S1	Th10/11	L4/5
**PSD Complications**										
Paraspinal abscess	yes	no	yes	no	no	yes	no	no	no	no
Psoas abscess	no	yes	yes	no	no	yes	no	no	no	yes
Destruction of a vertebral body	yes	yes	no	no	no	yes	yes	yes	yes	yes

m = male; f = female; PSD = pediatric spondylodiscitis.

**Table 2:** Laboratory data, microbiological findings, and treatment..

Patient	1	2	3	4	5	6	7	8	9	10
**Laboratory data at admission**										
ESR (mm/h)	40	44	95	34	125	3	n.a.	n.a.	n.a.	n.a.
WBC count (×10^9^/L)	17.1	6.9	10.7	10.2	16.8	6.2	14.7	15.3	6	7.5
CRP (mg/L)	4.3	48.2	9	7.6	2	4.6	14.6	11.7	9.9	<3
Hb g/dL	10.5	14.5	10.2	10.9	10.7	10.2	9.5	10.4	12.4	12.9
**Imaging**										
Sonography of the hip	yes	no	no	yes	yes	yes	no	yes	no	no
X-ray of the extremities /lumbar spine	yes	no	no	no	no	yes	no	yes	no	yes
CT	yes	no	no	no	no	no	no	no	yes	no
MRI	yes	yes	yes	yes	yes	yes	yes	yes	yes	yes
**Microbiological findings**										
Number of blood cultures	2	0	5	2	1	3	3	3	0	2
Blood culture result	sterile	n.a.	sterile	sterile	sterile	sterile	sterile	sterile	n.a.	sterile
CT-guided biopsy	yes	yes	no	yes	no	no	no	no	no	yes
Open biopsy	no	no	no	no	no	no	no	no	yes	no
Tests for tuberculosis	Quantiferon	no	Quantiferon	PCR + culture	Quantiferon	no	Quantiferon	no	no	PCR + culture
Biopsy result	sterile	*MSSA*	sterile	sterile	sterile	sterile	sterile	sterile	*MSSA*	*M. tuberculosis*
**Treatment**										
Surgery	no	no	no	no	no	no	no	no	yes	yes
Antibiotic i.v.	Cefotaxim Flucloxacillin	Flucloxacillin	(1) Clindamycin Cefuroxim, (2) Piperacillin/Tazobactam Metronidazol (3) Ciprofloxacin Clindamycin	Clindamycin Cefotaxim	Cefotaxim Flucloxacillin	Cefotaxim Flucloxacillin	Cefotaxim Flucloxacillin	Cefotaxim Flucloxacillin	Flucloxacillin Rifampicin	n.a.
Antibiotic p.o.	Amoxicillin/Clavulanic acid	no	Ciprofloxacin Clindamycin	no	Amoxicillin/Clavulanic acid	Amoxicillin/Clavulanic acid	n.a.	Amoxicillin/Clavulanic acid	no	Rifampicin Isoniazid Ethambutol Pyrazinamid
Total duration of antibiotic therapy (weeks)	6	6	6	3	6	6	min. 2 (Patient was transferred)	6	6	21 months
Duration of i.v. therapy (weeks)	3	3	4	3	2	2	min. 2	2	6	0
Duration of p.o. therapy (weeks)	3	3	2	0	4	4	n.a.	4	0	21 months
Duration from admission until initiation of therapy (days)	16	2	13	1	7	3	6	2	5	2

ESR = erythrocyte sedimentation rate (normal value < 20 mm/h); WBC = white blood cell count (normal value is age adapted); CRP = C-reactive protein (normal value < 5 mg/L); Hb = hemoglobin (normal value > 10 g/dL); CT = computed tomography; MRI = magnetic resonance imaging; i.v. = intravenous; p.o. = per os; n.a. = not applicable; n.a, *MSSA* = *Methicillin-Sensitive Staphylococcus aureus*; *M. tuberculosis* = *Mycobacterium tuberculosis*.

## Data Availability

The datasets generated during and/or analyzed during the current study are not publicly available but are available from the corresponding author on reasonable request.
